# Successful outcome of permanent maxillary incisor reimplanted after 30 hours of extra‐oral time—a case report with 5‐year follow‐up

**DOI:** 10.1002/ccr3.7721

**Published:** 2023-07-20

**Authors:** Ibadat Preet Kaur, Ashok Kumar, Mukul Kumar, Kanistika Jha

**Affiliations:** ^1^ ESIC Medical College and Hospital Alwar India; ^2^ Department of Pedodontics and Preventive Dentistry ESIC Dental College and Hospital New Delhi India; ^3^ College of Medical Sciences Bharatpur Bharatpur Nepal

**Keywords:** bioactive materials, delayed reimplantation, dental avulsion, regenerative endodontics, root‐resorption

## Abstract

**Key Clinical Message:**

Tooth reimplantation should be attempted in every possible case with thorough disinfection and impervious obturation. Integration of progressive innovations with recommended protocols can enhance complication free survival in intense situations.

**Abstract:**

The present case describes the unique successful outcome of a tooth reimplanted after the delay of 30 hours. A 21‐year old male patient presented with an avulsed #21 after a fall due to electric shock. It was reimplanted according IADT guidelines with the addition of specific irrigation sequence proposed for regenerative endodontic procedures during the endodontic treatment. The final obturation was accomplished by combination of Biodentine and BioRCS root‐canal sealer. Subluxated #11 developed symptoms at 6 months and was further managed using recommended endodontic procedure. Both teeth had complication free survival during 5 year follow‐up.

## 1 INTRODUCTION

Reimplantation after avulsion characterizes the reinsertion of an exarticulated tooth into its socket along with the associated procedures accomplished to secure it in the place for adequate healing with minimal complications. Depending upon the extra‐oral duration and storage conditions of an avulsed tooth, it is categorized as immediate or delayed reimplantation.^1^, ^2^ The procedure has variable success rates of 4%–50%, and the outcome is progressively decreased due to extended dry periods.^3^ Despite being associated with an increased risk of complications and failures, delayed reimplantation in comparison to immediate reimplantation is far more frequently accomplished treatment for a traumatic avulsion.^4^

Heimdahl and colleagues (1983) documented the first series exclusively describing the delayed reimplantations after extended extra‐alveolar time periods ranging from 6 h to 48 days in 18 teeth.^5^ The reimplantations with more than 24 h of extra‐oral time are scarcely reported in the literature with evidence of RRR/ankylosis at variable time intervals. (Table 1) The present case describes a 5‐year successful outcome of a permanent maxillary central incisor reimplanted after non‐physiological storage of 30 h. This report, to the author's knowledge, is the first of its kind, in using the standard disinfection protocol combined with the proposed method of bioactive obturation—as an additional attempt to limit the anticipated complications in excessively delayed reimplanted teeth.

**TABLE 1:** Summary of similar cases of delayed reimplantation with more than 24‐h extra‐oral dry time.

S. no.	Case no	Age (years)	Tooth/teeth avulsed	Extra–oral time	Treatment				Total follow‐up	Outcome	Author/year
					RCT	RF	RST	DR			
1.	1	15	11, 12, 13, 21, 22	10 days	EO	N	Y	Y	28 months	Considerable RRR on 12, 22. Slight RRR on the 21	Duggal MS et al. (1994)^6^
2.	2	15	11, 21	7 days	EO	Y	N	Y	8 years	Extensive RRR. Extraction Indicated	Cobankara FK, Ungor M (2007)^7^
3.	3	12	21	2 days	EO	N	N	Y	11 years	Extraction at 9 years. Replacement by implant at 11 years	Balleri P Et al. (2010)^8^
4.	4	12	21, 22	42 h	IO	N	Y	Y	7 months	Asymptomatic with signs of RRR	Ritwik et al. (2012)^9^
5.	5	13	11, 21	72 h	EO	N	N	Y	12 weeks	Asymptomatic with RRR and ankylosis	Ize‐Iyamu IN, Saheeb B (2013)^10^
	6	24	21	72 h	EO	N	N	Y	16 months	Asymptomatic without RRR and ankylosis	
6.	7	12	11	36 h	EO	N	Y	Y	24 months	Asymptomatic without RRR and ankylosis	Harris A et al. (2014)^11^
7.	8	8	21	27 h	EO (MTA obturation)	N	N	Y	18 months	Asymptomatic with RRR, ankylosis and infra‐occlusion	Savas S. et al. (2015)^12^
8.	9	8	21	4.5 days	EO (Portland Cement obturation)	N	Y	Y	18 months	Asymptomatic with RRR and ankylosis	Kolli NK et al. (2017)^13^
9.	10	10	11	26 h	IO	N	N	Y	10 years	Infraposition corrected by composite veneer. Asymptomatic with RRR and ankylosis.	Brunet‐Llobet L et al. (2018)^14^
10.	11	6	11	75 h	IO (with apical plug)	N	Y	Y	3 years	Asymptomatic without RRR and ankylosis	Vafaei et al. (2018)^15^
11.	12	11	11	6 days	EO	N	N	Y	1 year	Asymptomatic with progressive RRR and ankylosis	Gonçalves PSP et al. (2018)^16^
12.	13	14	11	More than 24 h for all five cases	N	N	N	Y	2 years for all five cases	I‐Severe IRR	Hasanuddin S, Reddy JS (2018)^17^
	14	09	11		EO	N	N	Y		II‐Severe IRR	
	15	10	11		EO	N	N	Y		III‐Complete RRR	
	16	12	11		EO	N	N	Y		IV‐Complete RRR	
	17	12	11, 21		EO	N	N	Y		V‐Complete RRR of both teeth, Extracted	
13.	18	21	11	More than 4 days	IO	N	N	Y (using i‐PRF)	1 year	Asymptomatic without RRR and ankylosis	Suresh N (2021)^18^

Abbreviations: DR, delayed reimplantation; EO, extra‐oral; IO, intra‐oral; IRR, inflammatory root resorption; N, no; RCT, root canal treatment; RF, retrograde filling; RRR, replacement root resorption; RST, root surface treatment; Y, yes.

## 2 CASE PRESENTATION

**FIGURE 1:**
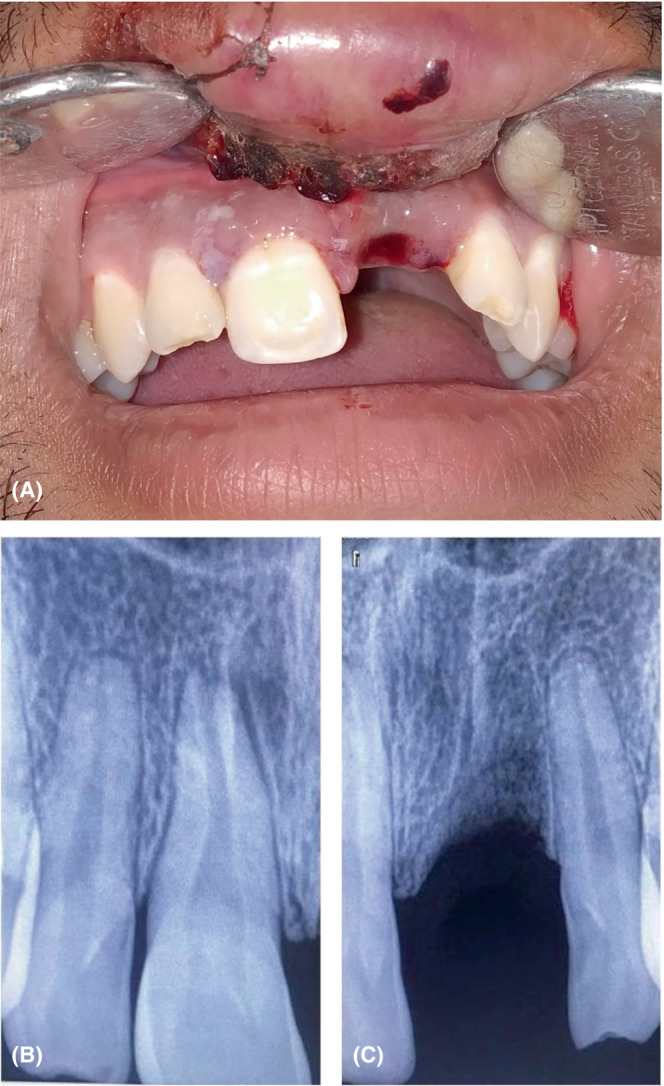
Pre‐operative—(A) Clinical Maxillary frontal view showing subluxated #11, empty socket of #21 and labial abrasion (B) IOPA of undisplaced #11 (C) IOPA of empty socket of# 21.

A 21‐year‐old Indian male patient presented with the complaint of loss of an upper tooth after a fall due to an electric shock after 30 h of injury. History revealed that loss of consciousness for 30 min and generalized bruises on the left side of body, also occurred due to injury. He was taken to a nearby hospital by his parents where following primary emergency care, an observational 24‐h admission for neurological assessment, was provided. No attempt for the reimplantation of the avulsed tooth had been made during the admission period. It was secured in a cloth piece for first 22 h and was later transferred to milk on the telephonic advice of a dental student. Extra‐oral examination recorded swelling and lacerations of the upper lip and abrasions on the nasal bridge and left malar region. Intra‐orally, the socket of avulsed #21 was filled with a blood clot (Figure 1A). Tooth **#**11 was tender on percussion with Grade‐II mobility while **#**12 and **#**22 had flattened incisal edges which were unrelated to the present injury. Electric pulp testing of the five maxillary and six mandibular anterior teeth recorded positive responses. The exarticulated tooth had an intact crown with a closed root apex and was carried in a container of milk. Radiographic examination revealed an undisplaced **#**11 (Figure 1) and an empty socket of **#**21 without any associated alveolar bone fracture (Figure 1C). It concluded the final diagnosis of subluxation for **#**11 and avulsion for **#**21. Available treatment options of prosthetic rehabilitation or tooth reimplantation with questionable prognosis were explained in detail to the patient, and written informed consent for the reimplantation of an avulsed tooth was taken.

Anesthesia was achieved with labial infiltration using 2% Lignocaine with 1:200000 adrenaline in the vestibular region of **#**21. The socket space was gently curetted to remove the coagulum and rinsed with 0.9% normal saline solution. The avulsed tooth was washed with normal saline, wiped with a sterile gauze, and repositioned in the hollow socket using light digital pressure. Both luxated **#**11 and reimplanted **#**21 were checked for alignment in occlusion and were finally stabilized with a flexible splint from 14 to 23 using a 28 gauge, stainless steel round wire and acid‐etch composite resin.

**FIGURE 2:**
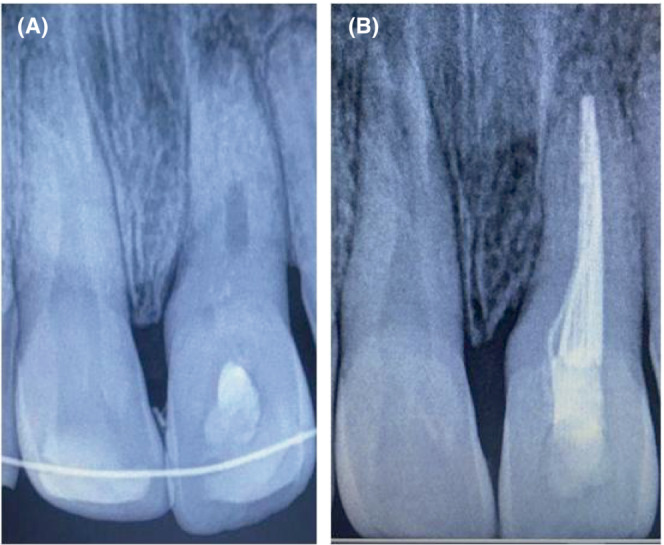
Peri‐operative (A) Immediate IOPA of reimplanted #21 with intracanal calcium hydroxide stabilized with composite splint (B) 4‐week IOPA of #21 immediately after obturation and splint removal.

The access cavity preparation of **#**21 was done at the same appointment, and pulp was extirpated in total using a barbed broach. Working length was measured electronically with the number 50 K file. The mechanical preparation was completed till no 70 K‐file using the hybrid technique with intermittent irrigation. The final irrigation sequence of 2.5% sodium hypochlorite (20 mL/canal, 5 min), 0.9% saline (20 mL/canal, 5 min) and 17% EDTA (20 mL/canal, 5 min) was followed. Canals were dried with paper points and intracanal medicament of a thick paste of calcium hydroxide powder in the aqueous base was placed (Figure 2A). The access cavity was sealed with high‐density GIC. The patient had already received a tetanus booster and three intravenous doses of the combination of amoxicillin + clavulanic acid (1000 + 200 mg) since the previous day; hence, oral dosage (500 + 125 mg TDS) of the same was prescribed for the next 6 days. Postoperatively soft diet, brushing after every major meal, and 0.12% chlorhexidine mouth rinses thrice a day along with regular weekly follow‐ups was also advised.

**FIGURE 3:**
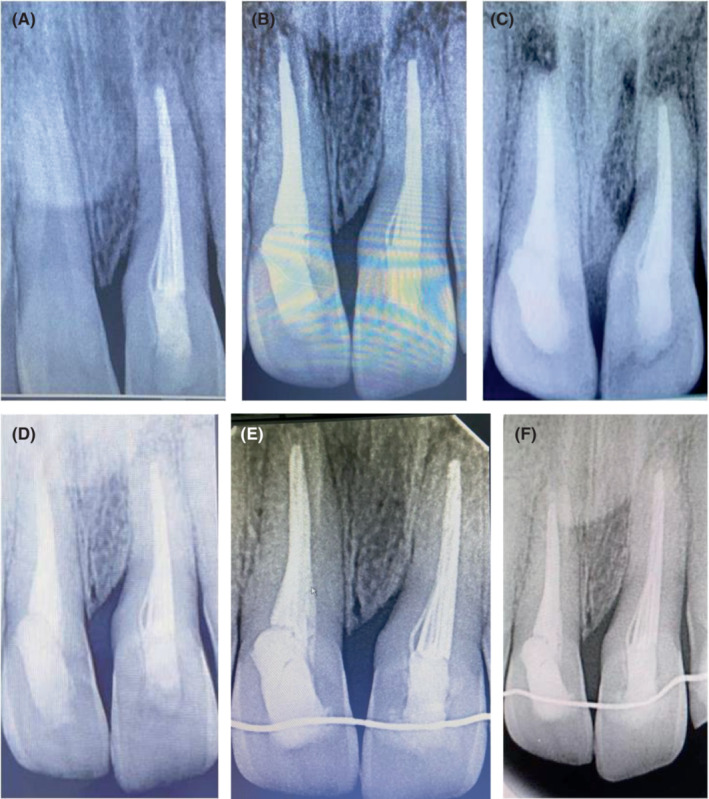
Radiographs of Follow‐up—(A) 6‐month (B) 1‐year (C) 2‐year (D) 3‐year (E)4‐year (F) 5‐year.

The tooth was re‐accessed at the second‐week follow‐up, and calcium hydroxide dressing was repeated in an aforesaid manner. At fourth week, the tooth was found to be asymptomatic and was planned for obturation at the same appointment using combination of 0.02 tapered Gutta‐percha points, Biodentine and Bio RCS. The apical tug‐back of the no. 70 master cone was confirmed clinically and radiographically at the measured root length. Biodentine was mixed according to the manufacturer's instructions, and it was applied apically and circumferentially along the walls with no. 60 Gutta‐percha point. The master cone was inserted, and conventional cold lateral compaction using Bio RCS and accessory cones was carried out to the extent that no more cones penetrated the coronal third of the canal. The final access cavity was sealed with light cure GIC layered by light cure resin restoration. At the simultaneous splint removal, reimplanted **#**21 recorded slightly excess than normal physiological mobility while subluxated **#**11 was stable and asymptomatic. The immediate post‐operative radiograph was recorded (Figure 2B), and further follow‐ups were scheduled for him. At 6‐month follow‐up, the presentation of **#**21 was consistent (Figure 3) whereas **#**11 was symptomatic and recorded a negative response to electric pulp testing. Endodontic treatment for it was commenced immediately with thorough chemico‐mechanical cleaning and placement of intermittent calcium hydroxide dressing for 21 days. The final obturation was done with 0.02 Gutta‐percha and resin based sealer using cold lateral compaction, and the access cavity was sealed in a way similar to **#**21.

**FIGURE 4:**
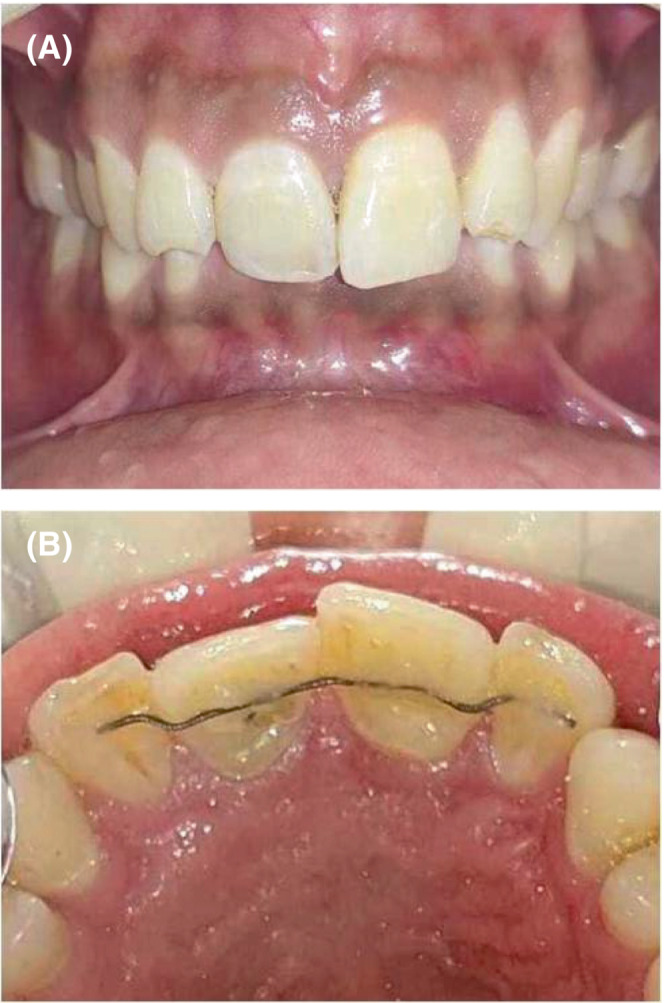
Clinical photographs at final Follow‐up—(A) 5‐year frontal view (B) 5‐year occlusal view.

The patient's periodic examination for 2 years documented stable presentation with the absence of any clinical or radiographic signs of root resorption or ankylosis (Figure 3B,C). Considering the persistent but stable Grade I mobility for the reimplanted tooth, a periodontal consultation was scheduled for him. However, his physical visit was precluded amidst the COVID‐19 and regular teleconsultation regarding the status of treatment was done. The 3‐year IOPA received through e‐consultation was also invariable (Figure 3D). The 4‐year physical follow‐up again documented the aforementioned observation, that is, an asymptomatic **#**21 with an unchanged persistent grade I mobility without any associated complications (Figure 3E). The 0.49 mm passive, co‐axial SS steel wire was bonded on the palatal surfaces of 12 to 22 after joint periodontal and orthodontic consultation. Both teeth were completely functional with the absence of any clinical (Figure 4A,B) and radiographic complications at 5 years (Figure 3F). The case progression is depicted as Timeline in Table 2.

**TABLE 2:** Timeline of case progression.

Time	Event	Clinical/radiographic assessment	Intervention
0	Patient reported after 30 h of injury.	Subluxation of #11 and Avulsion of #21 without any alveolar fracture confirmed. #21 had intact crown and closed apex #11 had positive response on EPT	Reimplantation of #21 followed by flexible splinting for both teeth. RCT of #21 using specified protocol commenced. Calcium hydroxide placed
+2 weeks	Physical visit	#11‐Asymptomatic and positive response on EPT #21‐Tenderness on percussion present	#21‐Calcium hydroxide dressing repeated
+4 weeks	Physical visit	#11‐Asymptomatic and positive response on EPT #21‐Asymptomatic with Grade‐I mobility	RCT of #21 completed using bioactive obturation Splint removed
+3 months	Physical visit	#11‐Asymptomatic, EPT not performed #21‐Asymptomatic with Grade‐I mobility No RRR or ankylosis	None
+6 months	Physical visit	#11‐Symptomatic, No response on EPT #21‐Asymptomatic with Grade‐I mobility No RRR or ankylosis	RCT of #11 accomplished
+1 year	Physical visit	#11‐Asymptomatic #21‐Asymptomatic with Grade‐I mobility No RRR or ankylosis	None
+2 years	Physical visit	#11‐Asymptomatic #21‐Asymptomatic with Grade‐I mobility No RRR or ankylosis	Oral Prophylaxis
+3 years	Telecommunication	#11‐Asymptomatic #21‐Asymptomatic No RRR or ankylosis	None
+4 years	Physical visit	#11‐Asymptomatic #21‐Asymptomatic with Grade‐I mobility No RRR or ankylosis	#21 reinforced with palatal fixed retainer
+5 years	Physical visit	#11‐Asymptomatic #21‐Asymptomatic No RRR or ankylosis	Oral Prophylaxis

Abbreviations: EPT, electric pulp testing; RCT, root canal treatment; RRR, replacement root resorption.

## 3 DISCUSSION

Immediate reimplantation within 5–20 min of extra‐oral dry time is globally accepted as the most appropriate emergency treatment for an avulsed tooth.^1^, ^2^ However, as in the present case, it is not always accomplished due to concomitant severe injuries, complex damage to the recipient sites and non‐availability of a dentist, or simply lack of awareness regarding the emergency procedures.^19^ A recent retrospective clinical study of 196 avulsed and reimplanted teeth, similar to previous studies, suggested non‐physiologic storage within 30 min as a critical time limit for the periodontal healing of replanted teeth.^20^ IADT currently sets the threshold extra‐oral dry time limit to 60 min for the PDL cell viability, regardless of whether the tooth has been stored in the medium or not.^2^ The patient had been admitted to a medical emergency for 1 day but unfortunately, no attempt for tooth reimplantation, even subsequent to critical care had been made there. He reported after 30 h of avulsion of **#**21 that had been transferred to milk after the dry period of 22 h. The prolonged extra‐socket time expectedly accelerates the onset and rate of proposed complications and reduces the final survival time of such teeth after reimplantation.^1^, ^2^ An attempt for reimplantation is still recommended even in extreme cases due to considerable benefits of saved natural tooth.^2^ Therefore, reimplantation of an avulsed tooth with minor modifications in accepted IADT protocol in accordance with literary evidence was accomplished for the present case.^1^

Anticipating future pulpal necrosis, endodontic treatment was started intra‐orally on the very first day. The extra‐oral root canal treatment is an optional recommendation according to IADT guidelines.^1^ The intra‐oral endodontic treatment was expected to provide the provision of better disinfection by utilizing the advantages of mentioned irrigation protocol combined with intracanal medicament. Hence, it was advocated for the present case. The combined circumferential and crown down approach was used with hand K‐files for complete mechanical cleaning of the wide root canal. It was supplemented with chemical disinfection using a modified irrigation protocol recommended for regenerative endodontic procedures. These biological procedures use minimal or no instrumentation technique and reckon entirely on the standard chemical sequence for complete disinfection of the root canal.^21^ As the critical treatment objective was to retain the avulsed tooth rather than preservation of apical stem cells, instead of recommended 1.5% concentration, 2.5% sodium hypochlorite followed by normal saline and final rinse of 17% EDTA was used for irrigation. Calcium hydroxide has an established antimicrobial, anti‐resorptive, and mineralization‐inducing ability in reimplanted teeth.^22^ It had been placed as an intermediate intracanal medicament instead of triple antibiotic paste or definitive obturation. Mineral trioxide aggregate (MTA) due to its favorable material characteristics and properties similar to calcium hydroxide is being proposed as a potential single‐visit root canal filling material for delayed reimplanted teeth.^12^, ^13^ Biodentine is a dentinal substitute formulated using active biosilicate (ABS) cement‐based technology having efficacy equivalent to MTA and the potential to overcome the drawbacks of both calcium hydroxide and MTA as root canal filling materials.^23^ BioRoot RCS incorporates the same ABS technology and is recommended to be used as a root canal sealer with a single cone or cold lateral condensation method.^24^ A significant decrease in fracture resistance of teeth obturated completely with calcium silicate types of cement in comparison when used in thin layers as pulp capping or apical plugs has been documented whereas filling the root canals with the MTA‐based sealers has not recorded any additional advantages.^25^ Hence to achieve maximum benefits and minimize risks, the final obturation was carried out with Biodentine coating for lateral and apical radicular walls combined with laterally condensed gutta‐percha and BioRoot RCS sealer.

External root resorption is a serious complication after delayed replantation of avulsed teeth that presents as inflammatory or replacement resorption of the root surface, with the latter being more frequent.^26^ Pulpal necrosis along with cemental damage stimulates a rapidly progressive inflammatory resorptive process resulting in early loss of the reimplanted tooth. Contrarily, loss of the protective barrier due to traumatic damage to cementum affecting more than 20% of the root surface and extensive PDL cell necrosis particularly after 60 min of dry storage results in slow‐progressing replacement resorption and ankylosis.^27^ A meta‐analysis reported the incidence of replacement and inflammatory root resorption to be 51.0% and 23.2%, respectively, in reimplanted avulsed teeth.^26^ Out of the 18 cases reporting the reimplantation after 24‐h period (Table 1), only single case^18^ has recorded the complication free periodontal healing, without any evidence of replacement root resorption or ankylosis after minimum follow‐up period of 1 year. A single available study considering the outcome of 38 teeth reimplanted after the specific extra‐oral duration of more than 24 h, documents the signs of replacement resorption in 71.1% of teeth within the follow‐up periods of 6–57 months.^28^ The largest and longest reported clinical follow‐up study of 400 teeth recorded ankylosis in 86.4% of teeth reimplanted after dry storage of 60 min. Additionally, 100% of teeth reimplanted after PDL removal were found to be ankylosed at various time intervals.^29^

Contradictory to previous evidence, an exceptional outcome with functional healing even after complete necrosis of PDL was found at a 5‐year follow‐up in the present case. It can be attributed to the complete mechanical debridement combined with the standard irrigation protocol that blocked the initial infectious processes. It is extremely significant in teeth with wide root canals, where incomplete instrumentation frequently results in the presence of tissue remnants in the dentinal walls.^30^ EDTA conditioning removes the smear layer and decalcifies dentin to a depth of 20–30 μm in 5 min.^31^ Biodentine and BioRCS are calcium releasing, antimicrobials with high alkalizing activity.^32^ They also have a superior sealing ability due to the formation of 5–15 μm tags of mineralized tissue in the dentinal tubules.^32^ The open dentinal tubules ensured greater penetration of irrigants and intracanal medicaments while the exposed dentin matrix further enhanced micromechanical sealing minimizing the detrimental effects of inflammatory factors subsequent to reimplantation. Additionally, the osteoinductive potential and inhibitory effects on the RANK/RANKL mediated odontoclast differentiation by bioactive materials further aided in the long‐term prevention of root resorption after delayed reimplantation.^33^

Ankylosis after delayed reimplantation is considered a preferred sequel in comparison to external inflammatory resorption as teeth may remain asymptomatic and serve the purpose for many additional years.^29^ The present tooth similar to the previous cases had also been reimplanted with the aim to achieve long‐term ankylosis. The Grade I mobility after splint removal was expected to be resolved with the onset of ankylosis. However, its persistence along with the radiographic examination suggested the functional healing of the reimplanted tooth till 4 years of clinical follow‐up. Lauridsen et al.^29^ suggested the association of absence of ankylosis with the non‐removal of residual PDL cells before delayed reimplantation. Since it is minimally possible after 30 h extra‐socket time, the authors propose that as the initial inflammation was constrained; surrounding PDL remnants from the alveolar bone populated in the favorable environment of the damaged root and served as the source for the stem cells for PDL regeneration. The fixed palatal retainer was placed after a multidisciplinary discussion to prevent any accidental functional trauma. Teeth replanted with a necrotic periodontal ligament are expected to become ankylosed within 3 to 5 years with initial signs being evident within 3 years.^27^ The apparent absence of ankylosis in the present case after 5 years of reimplantation diminishes the possibility of eventual ankylosis in the future. Therefore, the patient is scheduled for annual recalls to record the further long‐term outcome of the procedure.

The risk of ankylosis remains a critical factor influencing the ultimate long‐term outcome after similar delayed replantations.^27^, ^29^ Investigated therapeutic modalities to delay ankylosis have evolved from simple scrapping of root surface, to root surface treatments and most recently the use of stem cells for the regeneration of the lost PDL cells.^34^, ^35^ However, none of these is considered as a strict recommendation by IADT 2012 and even recently modified 2020 guidelines due to the presence of insufficient clinical trials.^1^, ^2^ Moreover, it might not be possible to practice them consistently due to the limited availability of resources. The main limitation of the present case is the omission of any additional anti‐resorptive surface treatment in the management protocol despite of the complete PDL necrosis. The outcome adds to the literature the benefits of thorough disinfection and impervious obturation using a simple, feasible, and established disinfection protocol combined with bioactive materials, in the success of excessively delayed reimplanted teeth. The inception can further be investigated at higher levels of evidence for more specific results.

## 4 CONCLUSION

Reimplantation should be attempted in every possible case in accordance with IADT guidelines. Delayed reimplantations after prolonged extra‐oral time are rare and predictably associated with poor prognosis. The successful outcome of present case after complete disinfection of reimplanted tooth using standard irrigation protocol followed by obturation with modern bioactive materials can have additional advantage in diminishing the expected long‐term complications of similar cases.

## 5 PATIENT'S PERSPECTIVE

According to patient, “he was completely hopeless at the first visit and came only on the insistence of his relative. The expectations increased after each visit and the success of the procedure is not less than a miracle for him and his family.”

## AUTHOR CONTRIBUTIONS

**Ibadat Preet Kaur:** Conceptualization; investigation; methodology; writing – original draft; writing – review and editing. **Ashok Kumar:** Investigation; methodology; resources; writing – review and editing. **Mukul Kumar:** Methodology; writing – review and editing. **Kanistika Jha:** Conceptualization; methodology; visualization; writing – review and editing.

## FUNDING INFORMATION

No financial aid had been received for the present case study.

## CONFLICT OF INTEREST STATEMENT

The authors have stated explicitly that no potential conflict of interest has been reported in connection with this article.

## CONSENT

Written informed consent was obtained from the patient to publish this report in accordance with the journal's patient consent policy.

## Data Availability

Data supporting this research article are available from thecorresponding author or first author on reasonable request.
